# Objectively measured sedentary behavior in preschool children: comparison between Montessori and traditional preschools

**DOI:** 10.1186/1479-5868-10-2

**Published:** 2013-01-03

**Authors:** Wonwoo Byun, Steven N Blair, Russell R Pate

**Affiliations:** 1 Department of Exercise Science, Arnold School of Public Health, University of South Carolina, Columbia, SC, USA; 2 Clinical Exercise Physiology, Human Performance Laboratory, Ball State University, Muncie, IN, USA

**Keywords:** Sedentary behavior, Preschool, Montessori, Accelerometer

## Abstract

**Background:**

This study aimed to compare the levels of objectively-measured sedentary behavior in children attending Montessori preschools with those attending traditional preschools.

**Methods:**

The participants in this study were preschool children aged 4 years old who were enrolled in Montessori and traditional preschools. The preschool children wore ActiGraph accelerometers. Accelerometers were initialized using 15-second intervals and sedentary behavior was defined as <200 counts/15-second. The accelerometry data were summarized into the average minutes per hour spent in sedentary behavior during the in-school, the after-school, and the total-day period. Mixed linear regression models were used to determine differences in the average time spent in sedentary behavior between children attending traditional and Montessori preschools, after adjusting for selected potential correlates of preschoolers’ sedentary behavior.

**Results:**

Children attending Montessori preschools spent less time in sedentary behavior than those attending traditional preschools during the in-school (44.4. min/hr vs. 47.1 min/hr, *P = 0*.03), after-school (42.8. min/hr vs. 44.7 min/hr, *P = 0*.04), and total-day (43.7 min/hr vs. 45.5 min/hr, *P = *0. 009) periods. School type (Montessori or traditional), preschool setting (private or public), socio-demographic factors (age, gender, and socioeconomic status) were found to be significant predictors of preschoolers’ sedentary behavior.

**Conclusions:**

Levels of objectively-measured sedentary behavior were significantly lower among children attending Montessori preschools compared to children attending traditional preschools. Future research should examine the specific characteristics of Montessori preschools that predict the lower levels of sedentary behavior among children attending these preschools compared to children attending traditional preschools.

## Background

The prevalence of childhood obesity in the U.S. has reached epidemic proportions in recent decades [1,2]. Importantly, this trend also has been observed among preschool children [3,4]. There is growing recognition that time spent in sedentary behavior (i.e., sitting, watching TV, and playing video games) is associated with an increased risk of childhood obesity [5-8]. However, levels of sedentary behavior have not been described well, especially in the preschool population, and the surveillance of sedentary behavior in preschool children is needed.

Approximately 5.1 million children are enrolled in preschools or child care centers in the U.S. [9], and the majority spend more than 6 hours per day in these settings [10]. It is important, therefore, to monitor levels of sedentary behavior in the preschool setting. However, quantifying levels of sedentary behavior in young children is challenging due to their intermittent movement patterns and inability to recall past behavior [11].

Our research team has described preschoolers’ sedentary behavior levels during preschool hours, using accelerometry and a direct observation system [12,13]. The majority of their time was spent in sedentary behavior during preschool hours (42.1 min/hr or >80% of the observations). It was also observed that preschooler’s sedentary behavior levels varied by school policies and characteristics [14,15]. However, those studies only included samples of children attending traditional preschools, and to the best of our knowledge no study has described the levels of objectively-measured sedentary behavior in children attending Montessori preschools, which have become popular in recent years [16,17].

The popularity of Montessori preschools likely reflects their unique approach in terms of children’s education. At least two-thirds of the preschool hours are devoted to opportunities for self-chosen/directed activities, [18] and children attending Montessori preschools are allowed to freely move about during the course of the day [19]. This likely provides more opportunities for ambulation compared to traditional preschools, [20] and may reduce the time spent in sedentary behavior. Therefore, we suspected that levels of sedentary behavior among children attending Montessori preschools might differ from those of children attending traditional preschools. The purpose of this study was to compare the levels of objectively-measured sedentary behavior in children attending Montessori preschools with those of children attending traditional preschools.

## Methods

### Study design

A cross-sectional study design was used. Preschool children were recruited from traditional and Montessori preschools in metropolitan Columbia, South Carolina. Trained data collectors recorded arrival times and departure times from the schools every day in order to distinguish in-school sedentary behavior from after-school sedentary behavior. Each child’s daily sedentary behavior data were summarized by time of day: in-school, after-school, and total-day.

### Participants

The participants were preschool children aged 4 years old who were enrolled in 8 traditional and 9 Montessori preschools. All traditional and Montessori preschools were identified according to following criteria: 1) the school is licensed as a child care center by the Department of Social Services, 2) the curriculum meets state standards, and 3) the teachers have degrees in early childhood education. Montessori preschools had to meet following additional criteria to be invited in the current study: 1) the school is accredited or a member of national Montessori associations and 2) the teachers have a certification of Montessori teaching. The number of participants per preschool ranged from 21 to 33 in traditional preschools, and from 19 to 62 in Montessori preschools. Children with missing data for study variables were removed, and the following data were available for analyses: in-school sedentary behavior (N=167 in traditional and N=164 in Montessori), after-school sedentary behavior (N=137 in traditional and N=135 in Montessori), and total-day sedentary behavior (N=137 in traditional and N=136 in Montessori). Written informed consent was obtained from children’s parents or guardians prior to collection of data. The study was approved by the Institutional Review Board at the University of South Carolina.

### Accelerometer protocol

The preschool children wore ActiGraph accelerometers (ActiGraph model GT1M, Shalimar, FL). The accelerometers were initialized to collect data in 15-second intervals (epochs) to account for the spontaneous activity patterns of preschool children. The accelerometers were attached to the child’s right hip (anterior to the iliac crest) using an elastic belt. Each child was instructed to wear the accelerometer for five consecutive school days (Monday – Friday), and their parents received information about how to wear and remove the accelerometer during after-school hours. Trained staff checked each child’s accelerometer at the beginning of each school day. If a child was not wearing the accelerometer upon arrival at preschool, a temporary monitor was provided to collect in-school sedentary behavior.

### Sedentary behavior

Accelerometers collect and store count data according to movement frequency and intensity. Cutpoints were applied to the count data to determine the time spent in sedentary behavior. In the present study, a cutpoint of < 200 counts/15 seconds was used to define sedentary behavior. This cutpoint was developed specifically for preschool children (3 to 5 years) [21]. Using each child’s wear time as the divisor, cumulative time spent in sedentary behavior was averaged on an hourly basis (min/hr). This was to take into account differences in the monitoring times of children on a given day, and therefore allowed for comparisons to be made across preschools. Sixty-minutes of consecutive zeros was considered as non-wear time [22-24]. Due to variations in actual in-school hours among preschools, children have worn the accelerometer for at least 50% of school hours to be included in the in-school and total-day analysis. For the after-school and total-day analysis, children had to wear the accelerometer for at least 4 hours during the after-school period. Days that children were absent from preschool and days on which total wear time was ≥18 hours (i.e., monitor malfunction) were excluded from the analysis because those days do not represent typical days. It has been suggested that three or more days of accelerometry monitoring provides reliable measures of accelerometry-derived sedentary behavior in preschool children (intraclass correlations ≥ 0.80), [25] thus children who had at least 3 valid days of in-school and total day sedentary behavior were included in the present study.

### Demographic and anthropometric characteristics

Children’s age, gender, race/ethnicity, and socioeconomic status (SES) were reported by a parent or guardian using a parent survey. Parent education level was measured as a surrogate indicator of socioeconomic status. Child’s participation in after-school sports program (i.e., number of times played per month) also was reported by a parent or guardian. Child’s weight was measured to the nearest 0.1 kg using an electronic scale, and height was measured to the nearest 1 mm using a stadiometer, after children removed their shoes and outer clothing. Body Mass Index (BMI) was calculated (kg/m^2^) from the averages of height and weight.

### Statistical analyses

Descriptive statistics (mean and SD; frequency and percent) for the participants were calculated according to activity monitored during the different times of the day. Independent sample t-tests and Chi-square tests were used to determine differences in demographic and anthropometric variables between traditional and Montessori preschools. The differences in time spent in sedentary behavior between children attending traditional preschools and those attending Montessori preschools were determined using mixed linear regression models that included age, gender, race, parent education level, BMI, accelerometer wear time (hours/day), and preschool funding type (private or public) as covariates. For after-school and total-day sedentary behavior, after-school sports participation was additionally included as a covariate. Preschool was included as a random effect in the mixed models to take into account correlations among children from the same preschool. Mixed linear regression models also were used to determine if the covariates influenced time spent in sedentary behavior. The following covariates were included as independent variables: school type (Montessori or traditional), age, gender, race, BMI, parent education level, preschool funding type, and child’s participation in after-school sports program. The pseudo-R2 was calculated to determine the fraction of variance explained by the model [26]. All data were analyzed using SAS version 9.2 (SAS Institute, Cary, NC, USA).

## Results

### Descriptive characteristics

Children attending Montessori preschools were predominantly white and more likely to have parents with higher education levels, compared to children attending traditional preschools (Table 1). Children attending Montessori preschools participated more frequently in organized sports than those attending traditional preschools (Table 1). BMI was slightly lower among the children attending Montessori preschools, compared to those attending traditional preschools. The average number of days and hours per day that the children wore accelerometers during the in-school, after-school, and total-day period were similar between children attending traditional preschool and those attending Montessori preschools (Table 1).

**Table 1:** Descriptive characteristics of participants, Mean ± SD or percent

**Characteristics**	**In-school Activity**		**After-school Activity**		**Total-day Activity**	
	**Montessori**	**Traditional**	**Montessori**	**Traditional**	**Montessori**	**Traditional**
N	164	167	135	137	136	137
Age (years)	4.4 ± 0.5	4.5 ± 0.4	4.4 ± 0.6	4.5 ± 0.3	4.4 ± 0.6	4.5 ± 0.3
Gender (%)						
Boys	49.4	52.6	48.9	53.2	49.0	53.2
Girls	50.6	47.4	51.1	46.8	51.0	46.8
Race (%)^*^						
African American	23.2	43.1	25.2	43.1	25.0	43.1
White	66.4	37.1	65.9	37.2	66.2	37.2
Other	10.4	19.8	8.9	19.7	8.8	19.7
Preschool setting (%)^*^						
Private^†^	64.6	34.7	61.5	42.6	61.5	42.6
Public^†^	35.4	65.3	38.5	57.4	38.5	57.4
BMI (kg/m^2^)^*^	15.9 ± 1.7	16.3 ± 2.0	15.9 ± 1.8	16.2 ± 1.9	15.9 ± 1.8	16.2 ± 1.9
Wear Time^‡^						
Number of Days	4.8 ± 0.6	4.6 ± 0.6	3.7 ± 0.5	3.7 ± 0.6	3.7 ± 0.5	3.7 ± 0.6
Hours per Day	5.8 ± 1.2	5.9 ± 1.1	6.4 ± 1.4	6.5 ± 1.5	12.2 ± 1.1	12.4 ± 1.3

*Significantly different between traditional and Montessori preschools (*P* <.05).

†Percentage of preschool children attending private or public preschools.

‡Number of days and number of hours that children wore accelerometers.

### Sedentary behavior between Montessori and traditional preschools

Overall, children attending Montessori preschools spent less time in sedentary behavior than those attending traditional preschools during the in-school period, after adjusting for age, gender, race, BMI, parent education level, preschool setting, and preschools (Table 2). Also, children attending Montessori preschools spent less time in sedentary behavior than those attending traditional preschools during the after-school and the total-day period (Table 2). The lower time spent in sedentary behavior in Montessori preschools also remained after further adjusting for child’s participation in after-school sports program (Table 2). Among all subgroups of children (gender, race/ethnicity, parental education level or preschool funding type) those attending Montessori preschools engaged in less sedentary behavior than those attending traditional preschools.

**Table 2:** Time spent in sedentary behavior in children attending Montessori and traditional preschool (Mean ± SE)

	**Sedentary Behavior (min/hr)**								
	**In-school**			**After-school**			**Total-day**		
	**Montessori**	**Traditional**	P^*^	**Montessori**	**Traditional**	P^*^	**Montessori**	**Traditional**	P^*^
Total group	44.4 ± 0.9	47.1 ± 0.9	0.03	42.8 ± 0.8	44.7 ± 0.7	0.04	43.7 ± 0.5	45.5 ± 0.4	0.009
Gender									
Boys	43.5 ± 0.9	46.0 ± 0.9	0.04	41.7 ± 1.1	44.3 ± 1.1	0.09	42.9 ± 0.7	44.8 ± 0.7	0.03
Girls	45.4 ± 1.0	47.8 ± 0.9	0.06	43.8 ± 0.9	45.5 ± 0.8	0.08	44.2 ± 0.6	46.1 ± 0.5	0.007
Race									
African American	43.0 ± 1.2	46.7 ± 1.3	0.02	42.8 ± 1.2	46.6 ± 1.2	0.01	42.8 ± 0.6	45.7 ± 0.6	0.003
White	44.9 ± 0.8	47.4 ± 0.8	0.01	41.1 ± 0.9	42.7 ± 0.8	0.09	43.8 ± 0.6	45.3 ± 0.6	0.02
Other	45.3 ± 1.5	45.3 ± 1.1	0.87	44.0 ± 2.9	43.8 ± 2.4	0.96	44.0 ± 1.2	44.8 ± 0.8	0.59
Parent education									
≤ College	45.6 ± 1.8	47.0 ± 1.3	0.51	42.1 ± 2.8	44.1 ± 1.3	0.52	43.2 ± 1.4	44.1 ± 1.1	0.55
> College	44.8 ± 0.6	47.2 ± 0.7	0.01	43.6 ± 056	45.8 ± 0.6	<.001	44.3 ± 0.4	46.4 ± 0.5	<.001
Preschool setting									
Private	44.5 ± 0.8	49.0 ± 0.9	<.001	41.5 ± 0.9	43.6 ± 0.9	0.008	42.7 ± 0.6	45.1 ± 0.4	0.002
Public	46.3 ± 1.5	45.8 ± 1.2	0.82	44.0 ± 1.5	45.8 ± 1.3	0.38	45.1 ± 0.5	45.6 ± 0.5	0.47

Estimated times spent in sedentary behaviors are least-square means and SE adjusted for age, gender, race, BMI, parent education level, accelerometer wear time, preschool setting, sports participation, and preschools as appropriated.

*P values for the difference between Traditional and Montessori preschool.

### Multivariate analyses for the prediction of sedentary behavior

School type and gender were found to be significant predictors of in-school, after-school, and total-day sedentary behavior (Table 3). Age and race were significant predictors of in-school sedentary behavior (Table 3). Parent education level was a significant predictor of total-day sedentary behavior, and preschool funding type was a significant predictor of after-school sedentary behavior. Total variances in sedentary behavior explained by the mixed model were 48%, 22%, and 34% for in-school, after-school, and total-day, respectively (Table 3).

**Table 3:** Results of multivariate regression analysis for prediction of sedentary behavior in preschool children

**Independent variables**	**Sedentary Behavior (min/hr)**								
	**In-school**			**After-school**			**Total-day**		
	**β (SE)**	***F***	***P***	**β (SE)**	***F***	***P***	**β (SE)**	***F***	***P***
Intercept	52.1 (3.12)	-	<.001	47.8 (4.59)	-	<.001	50.7 (2.72)	-	<.001
School type (0 = Traditional, 1 = Montessori)	−2.70 (1.22)	4.80	.03	−1.83 (0.87)	4.36	.04	−1.72 (0.64)	6.90	.009
Age	−1.01 (0.44)	5.19	.02	0.12 (0.69)	0.03	.77	−0.45 (0.40)	1.23	.26
Gender 0 = girls, 1 = boys)	−1.45 (0.41)	12.7	<.001	−1.94 (0.63)	9.40	.003	−1.47 (0.37)	15.7	<.001
Race (0 = White, 1 = African American)	−1.23 (0.56)	2.57	.03	0.90 (0.81)	1.03	.27	−0.71 (0.49)	1.19	0.15
(0 = Other, 1 = White)	0.17 (0.67)		.80	1.06 (1.03)		.30	0.04 (0.59)		0.95
(0 = Other, 1 = African American)	−1.06 (0.69)		.12	0.16 (1.01)		.87	−0.68 (0.62)		0.27
BMI	0.06 (0.11)	0.29	.59	−0.07 (0.18)	0.16	.69	−0.09 (0.10)	0.87	.35
Parent education (0 = ≤ College, 1 = > College)	0.54 (0.61)	0.77	.38	1.88 (0.93)	2.87	.09	1.28 (0.54)	5.50	.02
Preschool setting (0 = Public, 1 = Private)	−0.16 (1.24)	0.02	.89	−2.10 (0.90)	5.41	.03	−1.05 (0.53)	3.83	.05
After-school sports participation (0 = < 3 times/mth, 1 = ≥ 3 times/mth)	−0.07 (0.20)	0.13	.71	−0.20 (0.78)	0.07	.89	−0.53 (0.46)	1.32	.25
R^2^ (MCCC), %	0.48			0.22			0.34		

MCCC, maximum cross-correlation coefficient [26].

## Discussion

This is the first study to describe and compare levels of objectively-measured sedentary behavior between children attending Montessori and traditional preschools. We found that the average time spent in sedentary behavior was significantly lower among children attending Montessori preschools. Not only did these children spend less time in sedentary behavior while in school, they also spent less time in sedentary behavior while out of school. Our findings are of particular importance because they suggest that the Montessori education system needs to be studied carefully to determine the specific factors that facilitate lower time spent in sedentary behavior. In addition to the finding that school type was a significant predictor of sedentary behavior, we also observed that socio-demographic factors (age, gender, and parent education level), and preschool funding type predicted time spent in sedentary behavior in multivariate analysis. These findings suggest that socio-demographic characteristics and school-level characteristics (e.g., policies and environments) have a significant influence on preschoolers’ sedentary behavior. Both characteristics should be considered when developing interventions that aim to reduce the time preschoolers’ spend in sedentary behavior.

There is evidence that preschool policies and practices can influence preschoolers’ sedentary behavior. Previous studies found that children attending preschools with policies regarding sedentary opportunities (e.g., limiting time for prolonged sitting and TV/DVD viewing) spent significantly less time in sedentary behavior compared to those attending preschools without such policies [14,27]. The Montessori education system is based on a fundamental approach that encourages children to teach themselves, with teachers serving as assistants in the classroom [28]. Unlike traditional preschools, children in Montessori classrooms are not required to sit and listen to teacher-directed instructions, but are encouraged to choose and participate in individual or group activities [29]. This approach likely explains a proportion of variance in preschoolers’ sedentary behavior. Traditional preschools could consider if it is feasible to include aspects of this policy to reduce time spent in sedentary behavior.

Research also suggests that preschoolers’ sedentary behavior levels are affected by the physical and social environment of the preschool. Children attending preschools with environments that discourage sedentary behavior (e.g., fewer TVs and computers or greater classroom size) have been shown to spend less time in sedentary behavior while in school [15,27]. The Montessori preschool environments are based on the theory that “the best learning is active” and that children learn within “prepared environments” in which they can freely perform self-directed activities [29]. In general, the Montessori school classrooms are large and open-spaced to facilitate children’s movement [29]. As an example, Montessori preschool classrooms typically are equipped with sets of materials for light intensity physical activities (e.g., materials for sweeping, dusting, cleaning, and gardening), and children are regularly engaged in physical activities using those materials in school [28,29]. This could explain our observed difference in sedentary behavior during the in-school period.

An interesting observation in this study was that children attending Montessori preschools also spent less time in sedentary behavior out of school, compared to children attending traditional preschools. This out-of-school sedentary behavior difference persisted even after adjusting for child’s socioeconomic status and participation in after-school sports program. This finding is of particular interest because it has been hypothesized that children who are more sedentary (or active) during one part of the day would compensate at other times of the day, resulting in daily total activity levels that are constant [30]. Under the compensation hypothesis, environmental influences on children’s sedentary behavior is limited, and biological control of sedentary behavior is predominant [31]. However, our data do not support this hypothesis, but rather suggest that school-based interventions should be developed and implemented to reduce daily sedentary behavior in preschool children.

It is also possible that the difference in after-school school sedentary behavior could be due to a carry-over effect, whereby the Montessori school policies and environments also influence children’s sedentary behavior out of school. In general, Montessori preschoolers are encouraged to perform various types of light physical activity during their attendance at school [32]. Such activities include serving snacks, washing the floor, dusting tables, watering plants, and going outside to collect leaves [29,32]. Therefore, it is possible that children continue to perform these types of light physical activities outside of school. In addition, parents who send their children to Montessori schools are encouraged to limit the use of strollers and other carriers, and the children of parents who follow this encouragement may spend less time in sedentary behavior [32].

Alternatively, although we adjusted for numerous potential correlates of sedentary behavior in preschool children (e.g., child’s socio-demographic factors, BMI, and sports participation), [22,33,34] the observed difference in sedentary behavior out of school could be due to factors such as neighborhood environments (e.g., hills in neighborhood and crime/safety), [35,36] home environments and policy (e.g., number of TVs/computers, TV/computer in child’s bedroom, and parent rules on screen time), [37-40] and parental behaviors (e.g., parent screen time) [33,41]. Future research is required to explore whether Montessori preschool policies continue to reduce sedentary behavior after the child has left the school environment.

To our knowledge, only one previous intervention study has been designed to reduce sedentary behavior in preschool children [42]. However, that intervention was designed to reduce TV viewing at home, and did not intervene to reduce overall sedentary behavior in preschool children. In general, opportunities to move freely in the traditional preschool classroom are limited. This is likely due to early childhood educators being encouraged to employ formal curricula, which focus primarily on cognitive and language-oriented academic achievement [43,44]. However, this approach may not be optimal for raising academic achievement [45-47]. Research suggests that children attending Montessori schools have higher test scores in math and science compared to children attending traditional preschools [48,49]. Considering these data and our observations, it appears that the Montessori education system can be a strategy to reduce sedentary behavior, while also allowing for high academic achievement.

The current study had strengths and limitations that should be acknowledged. A major strength of this study was the use of an objective measure of sedentary behavior. Due to the poor recall ability and sporadic activity patterns of young children, assessing the time spent in sedentary behavior in preschoolers is difficult. We quantified, using accelerometers, the levels of sedentary behavior in preschool children across the in-school, after-school, and total-day period. In addition, our samples of preschool children were drawn from both private and public preschools. However, the generalizability of our findings may be limited because participants of this study were volunteers from preschools, and all participants were recruited from one geographic location in the southeast U.S.

## Conclusions

Results from this study showed that levels of sedentary behavior were significantly lower among children attending Montessori preschools compared to children attending traditional preschools. The lower levels of sedentary behavior were observed both in and out of school. These findings imply that the policies and environments of the Montessori preschool have the potential to influence the time preschoolers spend in sedentary behavior. Future research should be conducted to identify the specific policies and environmental factors of the Montessori preschool that discourage preschoolers’ sedentary behavior.

## Abbreviations

SES: Socioeconomic status; BMI: Body mass index.

## Competing interests

The authors declare no competing interests.

## Authors’ contributions

WB contributed to the concept and design, drafting, revision, interpretation of the data, and supervision. SNB and RRP contributed to the study hypotheses, refining the data analyses, interpreting results, drafting the manuscript, and revising it through multiple drafts. All authors have read and approved submission of the manuscript.

## References

[B1] Ogden CL Carroll MD Prevalence of Obesity Among Children and Adolescents: United States, Trends 1963–1965 Through 2007–2008 2010 National Center for Health Statistics

[B2] Ogden CL Carroll MD Kit BK Flegal KM Prevalence of obesity and trends in body mass index among US children and adolescents, 1999–2010 JAMA 2012 307 483 490 10.1001/jama.2012.40 22253364 PMC6362452

[B3] Ogden CL Carroll MD Curtin LR McDowell MA Tabak CJ Flegal KM Prevalence of overweight and obesity in the United States, 1999–2004 JAMA 2006 295 1549 1555 10.1001/jama.295.13.1549 16595758

[B4] Ogden CL Flegal KM Carroll MD Johnson CL Prevalence and trends in overweight among US children and adolescents, 1999–2000 JAMA 2002 288 1728 1732 10.1001/jama.288.14.1728 12365956

[B5] Jackson DM Djafarian K Stewart J Speakman JR Increased television viewing is associated with elevated body fatness but not with lower total energy expenditure in children Am J Clin Nutr 2009 89 1031 1036 10.3945/ajcn.2008.26746 19244374

[B6] Janz KF Burns TL Levy SM Tracking of activity and sedentary behaviors in childhood: the Iowa Bone Development Study Am J Prev Med 2005 29 171 178 10.1016/j.amepre.2005.06.001 16168865

[B7] Pratt C Webber LS Baggett CD Ward D Pate RR Murray D Lohman T Lytle L Elder JP Sedentary activity and body composition of middle school girls: the trial of activity for adolescent girls Res Q Exerc Sport 2008 79 458 467 19177947 10.1080/02701367.2008.10599512 PMC2701393

[B8] Steele RM van Sluijs EM Cassidy A Griffin SJ Ekelund U Targeting sedentary time or moderate- and vigorous-intensity activity: independent relations with adiposity in a population-based sample of 10-y-old British children Am J Clin Nutr 2009 90 1185 1192 10.3945/ajcn.2009.28153 19776141

[B9] Davis WD Bauman K School Enrollment in the United States: 2008 Population Characteristics 2011

[B10] National Center for Education Statistics Digest of education statistics, tables and figures 2001 2001 Washington DC

[B11] Bailey RC Olson J Pepper SL Porszasz J Barstow TJ Cooper DM The level and tempo of children’s physical activities: an observational study Med Sci Sports Exerc 1995 27 1033 1041 10.1249/00005768-199507000-00012 7564970

[B12] Pate RR Pfeiffer KA Trost SG Ziegler P Dowda M Physical activity among children attending preschools Pediatrics 2004 114 1258 1263 10.1542/peds.2003-1088-L 15520105

[B13] Pate RR McIver K Dowda M Brown WH Addy C Directly observed physical activity levels in preschool children J Sch Health 2008 78 438 444 10.1111/j.1746-1561.2008.00327.x 18651931

[B14] Dowda M Pate RR Trost SG Almeida MJ Sirard JR Influences of preschool policies and practices on children’s physical activity J Community Health 2004 29 183 196 15141894 10.1023/b:johe.0000022025.77294.af

[B15] Dowda M Brown WH McIver KL Pfeiffer KA O’Neill JR Addy CL Pate RR Policies and characteristics of the preschool environment and physical activity of young children Pediatrics 2009 123 e261 e266 10.1542/peds.2008-2498 19171578 PMC2632768

[B16] American Montessori Society American Montessori society and the Montessori movement 2011

[B17] Association Montessori Internationale Montessori movement 2012

[B18] Ramusch N Montessori in American: A history 1992 Portsmouth, NH: Heinemann

[B19] Montessori M Discovery of the child 1972 Ballantine Books

[B20] Montessori M Dr. Montessori’s own handbook 1914 New York: Stokes

[B21] Pate RR Almeida MJ McIver KL Pfeiffer KA Dowda M Validation and calibration of an accelerometer in preschool children Obesity (Silver Spring) 2006 14 2000 2006 10.1038/oby.2006.234 17135617

[B22] Byun W Dowda M Pate RR Correlates of objectively measured sedentary behavior in US preschool children Pediatrics 2011 128 937 945 10.1542/peds.2011-0748 22007010 PMC3208960

[B23] Ekelund U Luan J Sherar LB Esliger DW Griew P Cooper A Moderate to vigorous physical activity and sedentary time and cardiometabolic risk factors in children and adolescents JAMA 2012 307 704 712 10.1001/jama.2012.156 22337681 PMC3793121

[B24] Matthews CE Chen KY Freedson PS Buchowski MS Beech BM Pate RR Troiano RP Amount of time spent in sedentary behaviors in the united states, 2003–2004 Am J Epidemiol 2008 167 875 881 10.1093/aje/kwm390 18303006 PMC3527832

[B25] Byun W Blair SN Beets MW Dowda M Pate RR How many days of accelerometer monitoring predict sedentary behavior in preschoolers? [abstract] Med Sci Sports Exerc 2012 44 S480

[B26] Vonesh EF Chinchilli VM Pu K Goodness-of-fit in generalized nonlinear mixed-effects models Biometrics 1996 52 572 587 10.2307/2532896 10766504

[B27] Bower JK Hales DP Tate DF Rubin DA Benjamin SE Ward DS The childcare environment and children’s physical activity Am J Prev Med 2008 34 23 29 10.1016/j.amepre.2007.09.022 18083447

[B28] Montessori M The montessori mehtod 1988 Random House

[B29] Lillard AS The science behind the genius 2008 Oxford University Press

[B30] Fremeaux AE Mallam KM Metcalf BS Hosking J Voss LD Wilkin TJ The impact of school-time activity on total physical activity: the activitystat hypothesis (EarlyBird 46) Int J Obes (Lond) 2011 35 1277 1283 10.1038/ijo.2011.52 21407175

[B31] Wilkin TJ Mallam KM Metcalf BS Jeffery AN Voss LD Variation in physical activity lies with the child, not his environment: evidence for an ‘activitystat’ in young children (EarlyBird 16) Int J Obes (Lond) 2006 30 1050 1055 10.1038/sj.ijo.0803331 16801942

[B32] Montessori M The absorbent mind 1967 New York: Henry Holt

[B33] Kourlaba G Kondaki K Liarigkovinos T Manios Y Factors associated with television viewing time in toddlers and preschoolers in Greece: the GENESIS study J Public Health (Oxf) 2009 31 222 230 10.1093/pubmed/fdp011 19224946

[B34] Lee SJ Bartolic S Vandewater EA Predicting children’s media use in the USA: differences in cross-sectional and longitudinal analysis Br J Dev Psychol 2009 27 123 143 10.1348/026151008X401336 19829761 PMC2761041

[B35] Certain LK Kahn RS Prevalence, correlates, and trajectory of television viewing among infants and toddlers Pediatrics 2002 109 634 642 10.1542/peds.109.4.634 11927708

[B36] Norman GJ Schmid BA Sallis JF Calfas KJ Patrick K Psychosocial and environmental correlates of adolescent sedentary behaviors Pediatrics 2005 116 908 916 10.1542/peds.2004-1814 16199700

[B37] Hoyos CI Jago R Sociodemographic and home environment predictors of screen viewing among Spanish school children J Public Health (Oxf) 2010 10.1093/pubmed/fdq087 PMC3307230 21047871

[B38] Jago R Page A Froberg K Sardinha LB Klasson-Heggebo L Andersen LB Screen-viewing and the home TV environment: the European Youth Heart Study Prev Med 2008 47 525 529 10.1016/j.ypmed.2008.07.016 18722400

[B39] Roemmich JN Epstein LH Raja S Yin L The neighborhood and home environments: disparate relationships with physical activity and sedentary behaviors in youth Ann Behav Med 2007 33 29 38 10.1207/s15324796abm3301_4 17291168

[B40] van Sluijs EM Page A Ommundsen Y Griffin SJ Behavioural and social correlates of sedentary time in young people Br J Sports Med 2010 44 747 755 10.1136/bjsm.2008.049783 18812418

[B41] Songul YS Tugrul B Nacar N Tuncer M Yurdakok K Factors that affect television viewing time in preschool and primary schoolchildren Pediatr Int 2002 44 622 627 10.1046/j.1442-200X.2002.01648.x 12421259

[B42] Dennison BA Erb TA Jenkins PL Television viewing and television in bedroom associated with overweight risk among low-income preschool children Pediatrics 2002 109 1028 1035 10.1542/peds.109.6.1028 12042539

[B43] Farran DC Shonkoff JP, Meisels SJ Another decade of intervention for children who are low income or disabled: What do we know now? Handbook of early childhood intervention 2000 New York: Cambridge University Press

[B44] Gray SW Ramsey BK Klaus RA From 3 to 20: The early training project 1982 Baltimore: University Press

[B45] Davis CL Tomporowski PD McDowell JE Austin BP Miller PH Yanasak NE Allison JD Naglieri JA Exercise improves executive function and achievement and alters brain activation in overweight children: a randomized, controlled trial Health Psychol 2011 30 91 98 21299297 10.1037/a0021766 PMC3057917

[B46] Donnelly JE Lambourne K Classroom-based physical activity, cognition, and academic achievement Prev Med 2011 52 Suppl 1 S36 42 21281666 10.1016/j.ypmed.2011.01.021

[B47] Hillman CH Kamijo K Scudder M A review of chronic and acute physical activity participation on neuroelectric measures of brain health and cognition during childhood Prev Med 2011 52 Suppl 1 S21 28 21281669 10.1016/j.ypmed.2011.01.024 PMC3094734

[B48] Miller LB Bizzell RP Long-term effects of four preschool programs: ninth- and tenth-grade results Child Dev 1984 55 1570 1587 10.2307/1130027 6488964

[B49] Miller LB Dyer JL Four preschool programs: Their dimensions and effects Monogr Soc Res Child Dev 1975 55 1570 1587

